# Characterization of Semisweet and Sweet Wines from Kos Island Produced Traditionally and Conventionally

**DOI:** 10.3390/foods12203762

**Published:** 2023-10-13

**Authors:** Adriana Skendi, Stefanos Stefanou, Maria Papageorgiou

**Affiliations:** 1 Department of Agricultural Biotechnology and Enology, International Hellenic University, 1st Km Dramas-Mikroxoriou, 66100 Drama, Greece; 2 Department of Food Science and Technology, International Hellenic University, P.O. Box 141, 57400 Thessaloniki, Greece; 3 Department of Agriculture, International Hellenic University, P.O. Box 141, 57400 Thessaloniki, Greece

**Keywords:** sweet wines, ICP–OES, phenolic content, antioxidant activity, oenological parameters, natural grape dehydration

## Abstract

Eight wines, four semisweet rosé and four sweet red, produced on Kos Island in Greece, were analyzed. Wines produced following different winemaking procedures were characterized based on their physicochemical parameters, total phenolic content, antioxidant activity, and chromatic properties. Moreover, their elemental composition was studied with ICP–OES. Differences were observed among the measurements performed. All of the samples were below the levels set for SO_2_ content. The sweet red wines had higher alcoholic strength than semisweet rosé ones, and were characterized by a higher yellow proportion. The vinification process significantly affected SO_2_ levels, phenolics, and antioxidant activity. The red wines were high in Na content, with one sample exceeding the level set by OIV (International Organization of Vine and Wine). The levels of all the other elements related to quality (Fe, Cu, Zn) or safety (Pb, Cd) were far below the limits set. Rosé wines contained less Mg, but were higher in Na than the red ones. The obtained data suggest that sweet and semisweet wines produced with traditional procedures are safe and of high quality, holding antioxidant capacity beneficial to health. The information reported contributes to a better understanding of these types of wines.

## 1. Introduction

On the island of Kos, home of Hippocrates, the father of modern medicine, viticulture, and production of wine continued for four millennia. Kos is an island of Dodecanese (36°51′ N 27°14′ E) in the eastern segment of the southern Aegean region, considered a PGI (protected geographical indication) zone since 2008 (File number: PGI–GR–A0981, eAmbrosia Database [1]). All vineyards are positioned at altitudes exceeding 20 m, with most of the traditional vineyards planted on the northern slopes of Mount Dikeo, which rises in the center of the island. The temperature difference between day and night during the summer months particularly favors ripeness and a high content of pigments of red wines. Sunshine lasts for 2990–3000 h. The effect of the land and sea breeze is also crucial, as the vineyards at the foot of Dikaio mountain can offer a cooler environment owing to the slope of the land and orientation, especially on hot days, thus enhancing photosynthesis. In addition to local dry wine under commercial labels, semisweet and sweet wine are produced on the island using traditional methods. The combination of the above-mentioned special climatic conditions with the soil characteristics of the island, the grape varieties cultivated, the cultivation method, and the winemaking techniques applied contribute to the special quality characteristics of PGI Kos wines.

A variety of methods from traditional to modern ones are involved in the winemaking of sweet/semisweet wines produced on the island of Kos. Some of them involve extended ripening, where grape berries are left on the vine (on-vine drying), while others apply sun-drying after harvesting (off-vine drying) so that berries can lose most of their water without developing mold, leading to a grape-must high in sugars. Moreover, other methods that involve must volume reduction by heating or the addition of honey are also applied to increase the sugar content in the must.

Generally, the dehydration process affects different wine compounds, resulting in variations in the quality characteristics of sweet wines [2,3,4,5]. Among them, the content of phenolic compounds is the most prominent, since their variation contributes to multiple wine quality parameters such as color, sensorial quality, and antioxidant capacity [6]. Although phenolic content depends on the variety and maturity of grapes, as well as other factors affecting berry development (i.e., soil composition, geographical location, sun exposure, and weather conditions) [7,8,9], procedures related to the vinification process, including dehydration [4,5], affect it to a great extent. Moreno, Cerpa-Calderón, Cohen, Fang, Qian, and Kennedy [3] reported an increase in proanthocyanidin levels with dehydration and extended ripening, with dehydration being the most significant factor. Some phenolic compounds are strongly related to the formation of pigments (i.e., anthocyanins) that govern changes in the wine color. Chemical parameters such as pH and total acidity are also involved with the abovementioned changes; thus, their monitoring is crucial for maintaining wine quality. It has been reported that acidity increased during drying [5]. On the other hand, high pH values are noted to negatively influence the color and aroma, in addition to the increase in the microbial risk for the wines [10].

The literature reported an increase in the browning index and total antioxidant activity during the off-vine drying process [4,5]. Moreno, Peinado, and Peinado [2] noted a high correlation between antioxidant activity and sugar concentration, suggesting that the Maillard reaction contributes in a significant way to the formation of brown pigments, as well as to the increase in the antioxidant activity of the musts of sun-dried grapes. This fact implies a higher beneficial effect on human health if moderate quantities of sweet wines from sun-dried grapes are consumed. The use of SO_2_ limits the microbial risk and counteracts oxidative reactions (enzymatic and/or chemical) in wine, avoiding organoleptic alterations that favor the release of phenolics in the wine [11]. Nevertheless, adverse health reactions and environmental concerns related to the addition of SO_2_ have created a new health trend toward wines low in sulfites. In addition to different emerging winemaking trends [12] to meet consumers’ needs, a trend to adopt traditional winemaking processes or in combination with modern ones is also observed.

The elemental composition plays an important role in the quality of wines. Elemental analysis is used for the categorization of wines, as well as to authenticate their origin [13,14,15,16,17]. Metals considered as macroelements in wine (K, Ca, Mg, and Na) have great importance during fermentation by regulating pH and ionic balance, and are important for yeast redox processes of yeast metabolism. The microelements Fe, Cu, Zn, and Mn are found in lower amounts, and are essential to promote yeast enzymes [18]. Thus, metals affect alcoholic fermentation and influence the stability, color, and clarity of wines. Some of the elements must be controlled to avoid quality deterioration [19]. Modification of elemental composition is possible with the use of additives or during fining [19,20]. Moreover, the wine is constantly under monitoring. The elemental analysis is a prerequisite of legislation that limits the presence of some metals due to their toxic effect on the human body in the case of excessive intake. These elements are usually found in trace amounts and could be a result of environmental pollution, but also a result of cross-contamination during winemaking [18].

A limited number of articles in the literature deal with the characterization of sweet wines [21,22,23,24,25,26,27,28], and among them, only a few examine elemental composition. Thus, it is difficult to form conclusions about wine quality when little is known about the chemical and elemental composition, especially when considering differences in the vinification process applied in different countries/regions. Frías, Conde, Rodríguez-Bencomo, García-Montelongo, and Pérez-Trujillo [22] reported that sweet wines derived from overripe grapes differ in the chemical composition (except for sodium and iron) when compared to dry wines, while sweet wines from grapes of a similar ripening state do not. Until now, no research has been reported on sweet and semisweet wines from Kos Island, if taking into account the long history of traditional vinification procedures applied locally.

The majority of existing research focuses on dry wines (either red or white), while research papers on sweet and semisweet wines aiming at the determination of their chemical and elemental composition are quite rare in the literature, and are mostly focusing on the aromatic profile of sweet wines [29,30,31]. Thus, this work aims to cover this gap in the literature, providing more information about the enological characteristics of sweet and semisweet wines in relation to the vinification procedure and the specific terroir that needs to be reconsidered in the light of climate change. This research represents a first attempt to describe sweet wines from Kos Island and try to connect their quality attributes with the winemaking techniques applied. The obtained results can broaden the knowledge about sweet and semisweet wine fingerprinting.

## 2. Materials and Methods

### 2.1. Samples

A total of eight wine samples from Kos Island (Greece) were studied, all belonging to the production year 2020. Sample details and codification are reported in Table 1. Table 1 reports that sweet and semisweet wines (labeled based on OIV standards [32]) can be classified into four vinification processes (P1, P2, P3, P4): P1 includes samples S1, S2, and S3, where fermentation of must from sun-dried grapes is stopped artificially by cooling to a temperature that inhibits yeast growth; P2 includes sample S4, where fermentation of must from sun-dried grapes stops naturally; P3 includes samples S5 and S6, where fermentation of grape must with added honey stops naturally; and P4 includes samples S6 and S7, where the grape must was concentrated with cold cooked must (CCM) and fermentation stops naturally.

### 2.2. Chemicals and Reagents

Folin–Ciocalteu reagent and sodium hydroxide (HPLC grade) were purchased from Chem-Lab NV (Zedelgem, Belgium), whereas DPPH (2,2-diphenyl-1-picrylhydrazyl) was from Sigma Aldrich (St. Louis, MO, USA). Gallic acid, used to prepare the calibration curve for the determination of phenolics, and Trolox ((S)-(−)-6-hydroxy-2,5,7,8-tetramethylchroman-2-carboxylic acid), used to determine antioxidant activity, were obtained from J&K Scientific GmbH (Pforzheim, Germany). Sodium carbonate was from Merck KGaA (Darmstadt, Germany). All of the other chemicals used were of analytical grade. Water used to make dilutions and solutions, as well as methanol, were of HPLC (high-performance liquid chromatography) gradient grade from Chem-Lab NV (Zedelgem, Belgium).

For elemental analysis, single certified element standard solutions (1000 mg/L) of trace elements Arsenic (As), Cadmium (Cd), Lead (Pb), Nickel (Ni), and Chromium (Cr), microelements Iron (Fe), Copper (Cu), Zinc (Zn), and Manganese (Mn), and macroelements Potassium (K), Calcium (Ca), Magnesium (Mg), and Sodium (Na) were obtained from Sigma Chem (St. Louis, MO, USA). All glassware and plastic containers used for elemental analysis were first soaked in nitric acid (10%), and then washed properly with ultrapure water in order to avoid cross-contamination. The rest of the chemicals and reagents used were of reagent grade.

### 2.3. Chemical Analysis of Wines

Before all analyses, the obtained wine samples were centrifuged (5000 rpm for 10 min). The chemical analyses were performed using the analytical methods described in the Compendium of International Methods of Analysis of Wines and Musts [33]. Total acidity (TA) was assessed by titration using a sodium hydroxide solution (0.1 M) OIV–MA–F1–05 OIV–MA–AS313–01, and the results were expressed as g/L of tartaric acid. Wine samples were made alkaline by a suspension of calcium hydroxide, then were distilled and finally measured for alcoholic strength by the volume of the distillate (vol.%) as recommended by the distillation method OIV–MA–AS312–01A. The total and free contents of sulfur dioxide (SO_2_) were determined by the titrimetric method OIV–MA–AS323–04B.

### 2.4. Wine Color Determinations

After centrifugation (10 min, 5000 rpm) to remove any suspended particles, absorbance was read at 420, 520, and 620 nm in a UV–VIS spectrometer (Helios alpha, Thermo Fisher Scientific, Waltham, MA, USA) to measure color characteristics of wines according to Glories [34]. The wine color intensity was calculated as the sum of the three measured absorbances, whereas wine hue was defined as the absorbance ratio at 420 and 520 nm. Moreover, the chromatic structure of wine was evaluated as the relative percentage (contribution in %) of each of the three measured components (420 nm yellow, 520 nm red, 620 nm blue) to the total color (sum of them).

### 2.5. Determination of Phenolics and Antioxidant Capacity

For the determination of total phenolic content (TPC) and DPPH antioxidant capacity, samples were centrifugated (5000 rpm, 10 min) to remove any suspended particles and prevent haze formation during measurements. Wine samples were diluted appropriately with deionized water. The TPC was determined using the Folin–Ciocalteu method, and the results are expressed as micrograms of gallic acid per mL (μg GAE/mL). The antioxidant activity of wines was assessed by DPPH assay, and the results were reported as micrograms of Trolox equivalent per mL (μg TE/mL). Both methods have been previously described in more detail [35].

### 2.6. Elemental Analysis by ICP–OES of Wines

Alcohol was removed from the centrifuged (10,000 rpm, 10 min) wine samples by heating in a water bath until about 40% volume reduction. The evaporated amount was substituted with ultrapure water and after that, samples were diluted with 8% nitric acid aqueous solution in order to minimize the signal-suppressing effects of the organic constituents of wines, as reported in Skendi, Papageorgiou, and Stefanou [35]. According to Drava and Minganti [36], this procedure allows detection at a concentration range of ppb. Three replicates were prepared for each wine sample and analyzed in duplicate.

Concentrations of macroelements (K, Ca, Mg, Na), microelements (Fe, Cu, Zn, Mn), and trace elements (Cd, Pb, Ni, Cr, As) were determined by inductively coupled plasma optical emission spectrometry (ICP–OES) (Perkin Elmer, 8300DV, Waltham, MA, USA). This instrument was equipped with a time-of-flight analyzer, which guarantees almost simultaneous types of measurements for the whole mass spectra. The operating conditions for the multielemental analysis of wines on the ICP–OES spectrometer were as follows: the carrier gas used was Argon, plasma flow rate 10 L/min, auxiliary gas flow rate 0.6 L/min, nebulization gas flow rate 0.35 L/min, generator power 1500 W, nebulizer concentric quartz. Selected wavelengths (nm) for the elements are as follows: Cd (228.802), Pb (220.353), Ni (231.604), Cr (267.716), As (188.979), Fe (238.204), Cu (327.393), Zn (206.200), Mn (257.610), K (766.490), Ca (317.933), Mg (285.213), Na (589.592). The concentration of elements was determined in the axial plasma position.

The working standard solutions for calibration (three-point) were prepared daily by diluting the purchased primary standard solutions, using the wine matrix in a procedure similar to the standard addition method. Blank consisted of 2.0% nitric acid in ultrapure water. The method was developed following the IUPAC guidelines [37]. More about calibration, reproducibility, and accuracy, as well as limits of detection of samples, is reported by Skendi, Papageorgiou, and Stefanou [35]. The detection limit (μg/L) for the studied compounds are as follows: 0.8, 2.7, 1.8, 0.9, and 4.2, for trace elements Cd, Pb, Ni, Cr, and As; 9.7, 1.6, 5.9, and 2.6, for microelements Fe, Cu, Zn, and Mn; 63.1, 79.3, 10.0, and 15.0, for macroelements K, Ca, Mg, and Na, respectively. The results obtained were analogized with the maximum permissible limit (MPL) set by OIV international standards, as follows: Na 80 mg/L, Cu 1 mg/L, Zn 5 mg/L, Cd 0.01 mg/L, Pb 0.15 mg/L, and As 0.2 mg/L [32].

### 2.7. Statistical Analysis

Each wine was analyzed in triplicate, and the results were expressed as mean ± standard deviation. One-way ANOVA followed by Duncan’s post hoc test was performed to detect significant differences between wines. Associations among the parameters measured were estimated by Pearson correlation. SPSS Statistics 25.0 software (SPSS Inc., Chicago, IL, USA) was used to perform the abovementioned tests.

Principal component analysis (PCA) using the pairwise estimation method was also applied to the set of data to detect the relationship between variables and observations. JMP^®^ Pro 14.3.0 (SAS Institute Inc., Cary, NC, USA) software was used to perform PCA. In all of the performed statistical tests, a significance level of *p* < 0.05 was applied to note differences among the samples.

## 3. Results

### 3.1. Variation of Physicochemical Properties of Wines

Both pH and total acidity (TA) are considered indicators of wine quality, since they affect wine stability. Significant differences were observed among the samples (Table 2). No significant differences were observed between the red and rosé samples in pH and acidity values. The highest and the lowest pH values were observed in sweet red samples S7 and S8, respectively, whereas the respective TA values were found in sweet red S8 and semisweet rosé S2. There was a significant (at the 0.05 level two-tailed) negative correlation (−0.556) between the pH and TA. Statistical analysis revealed that the process does not affect the pH and TA values of the samples. This behavior could be due to acidity adjustment made in the case of P1 of P2, and differences in grape must composition derived from employment of different grape varieties and cultivation regions.

Sulfur dioxide (SO_2_) is commonly used in the winemaking process [38] in amounts that vary depending on the country’s regulations. It is considered an excellent preservative against microbial spoilage besides prevention of oxidation. According to the O.I.V., the legal limit for SO_2_ is less than 300 mg/L for wines with sugar content > 4 g/L, while for sweet wines it is less than 400 mg/L. Many other countries have more strict rules about the amount of SO_2_ in the final products, further limiting the permissible amount of SO_2_. These limitations are set due to potential adverse actions of SO_2_ such as intolerances and allergic reactions of a threshold varying between 5 and 200 mg/L SO_2_ [38]. In our study, the amount of total SO_2_ varies from 15.60 to 192.30 mg/L, with red wines showing lower values than the rosé ones. The observed values are much lower (<192.3 mg/L for rosé and <76.2 mg/L for red) than the limit of 250 for white and rosé with ≥5 g/L sugars, and 200 mg/L for red with ≥5 g/L sugars set in the EU [39] legislation. This fact is in accordance with the literature that suggests the addition of higher levels of sulfite in white and rosé wines, and much higher in sweet wines compared to red wines, because the latter are more protected due to higher natural antioxidant content. The lowest levels of SO_2_ are observed in samples S7 and S8 with no added SO_2_; even if there are no added sulfites, yeast has the ability to naturally produce SO_2_ during fermentation. This amount can vary from 10 to 50 mg/L [38]. The above samples (S7 and S8), together with S5, are far below the EU limit and the value of 50 mg/L [40]. Comparable to the pattern of total SO_2_, semisweet rosé wines showed significantly higher free SO_2_ values than sweet red. Free SO_2_ values varied from 2.95 to 65 mg/L.

The presence of SO_2_ is linked to some undesired effects in wine, such as fermentation delays or prevention of malolactic fermentation, creation of unpleasant flavors [11], and adverse reactions in human health [41]. Winemaking processes used for sweet red wines do not produce any change in the quantity of free and total SO_2_. On the other hand, for the semisweet rosé, P2 showed higher free SO_2_ levels, but similar total SO_2_ levels than P1. As expected, sample P4, with no added sulfites, showed the lowest total SO_2_ content.

The alcohol content was significantly higher In the red samples than in the rosé, with a difference in the mean values of greater than 1% vol. (Table 2). The amount of alcohol in this study was greater than 12%, and much higher than that reported for naturally sweet wines by Garnacha Tintorera [26]. One rosé wine (S3) showed a very high alcohol level (14%), similar to that of sweet red S7 sample. In sample S3, as well as in S1 and S2, the initial quantity of sugars allows fermentation to achieve a high alcohol level. So, by stopping fermentation artificially (by cooling), the producer regulates the final alcohol level. As expected, samples S5 and S6 show similar alcoholic strength since the fermentation begins with the same sugar content, and the same amount of honey is added and the same wine making procedure is followed. The winemaking procedure P4 resulted in a significantly higher alcohol level than P3. The alcohol level produced by applying process P3, with honey addition, was lower than that of P4. Similarly, Jastrzębska [42] reported lower alcohol content in wine produced with honey addition, compared to the ones without.

Significant correlations were observed among the chemical parameters of wines. The pH was negatively correlated with TA (−0.556, *p* < 0.05). On the other hand, TA was positively (0.584, *p* < 0.05) correlated, while free SO_2_ was negatively (−0.517, *p* < 0.05) correlated with the alcohol content. Moreover, it is observed that altitude cannot be associated with any of the other parameters, pH, TA, and alcohol strength.

### 3.2. Variation of Chromatic Parameters

**Figure 1:**
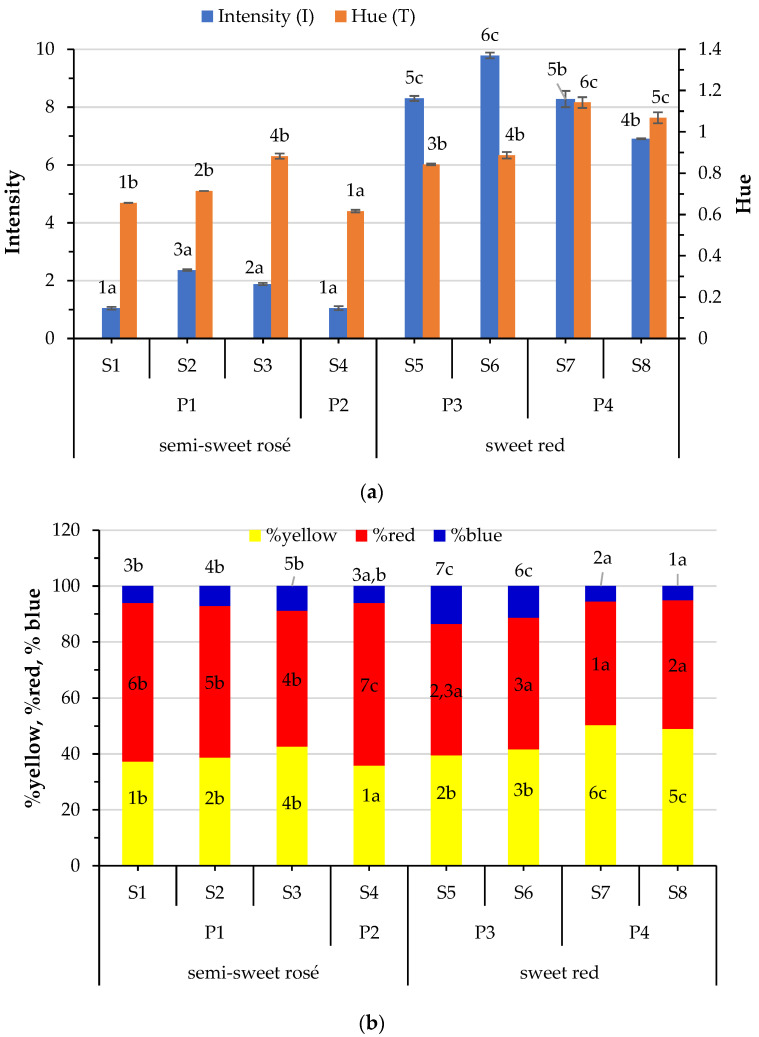
Variation of chromatic properties of wines according to the type of wine (semisweet rosé and sweet red) and process (P1–P4): (**a**) color intensity and hue; (**b**) the percentage of yellow, red, and blue hues. Values are means of three replicates. Means of samples with any similar superscript number for the same parameter reveal that samples do not differ significantly (*p* < 0.05) by Duncan’s multiple range test. Means of samples with any similar superscript letter for the same parameter reveal that processes do not differ significantly (*p* < 0.05) by Duncan’s multiple range test. Sorted from the lowest to highest values, where “a” was the lowest.

The chromatic parameters of the examined wines are presented in Figure 1. In addition to the hue and the intensity (Figure 1a), yellow, red, and blue hue contributions to the wine color are determined (Figure 1a). Wine color intensity depends on pigmentation, and varies mainly based on the grape variety and type of wine. Wine hue is partially affected by the pH level, and is an indicator of color development during ageing. The highest value of color intensity was observed in S6 sweet red wine, and the lowest value was recorded in semisweet rosé samples S1 and S4. Hue varied among the samples with sample S4 showing the lowest value and S7 the highest. As expected, higher color intensity and hue were recorded in red wines. Differences observed between S5 and S6, and S7 and S8 can be attributed to the differences in the varietal blend, since the applied vinification procedure was the same. The same is true for the semisweet rosé wines.

The wine chromatic structure differs among the samples. The red component (44.10–58.09%) was the major one, followed by yellow and then blue. Maillard reactions developed during sweet wine production can promote yellow and brown shades depending on the pH, time, and temperature applied, altering the final color of the wine. Sweet red wines exhibited yellow values much higher than that of rosé, while the opposite was observed for the red component values. Although there are differences in the blue component among the samples, no difference was observed between sweet red and semisweet rosé wines. The presence of honey in samples S5 and S6, or exposure to the high temperatures during must concentration in samples S7 and S8, could be the reason for the substantially increased hue of the sweet red wines. A decrease in water activity during concentration and high temperature accelerates the Maillard reaction, which produces not only brown-colored polymers, but also simple derivatives of furan that increase the brown color. The highest hue values and yellow pigments in samples S7 and S8 produced by process P4 could be explained by oxidation and condensation reactions in the must, these reactions obviously being markedly favored by the high temperature applied during concentration of the must compared to those during sun-drying.

The color of the wine is affected by pH because it affects the equilibrium between different forms of anthocyanins, and favors certain polymerization reactions or the condensation of red wine pigments [43]. In the present study, no correlation was observed between any parameter of the color of the studied semisweet and sweet wines and the pH or TA. Oxidation and color alteration by the presence of SO_2_ are reported in the literature [44]. Indeed, intensity and hue of wines were negatively correlated with free SO_2_ (−0.655 and −0.680, respectively, *p* < 0.01) and total SO_2_ (−0.699 and −0.520, respectively, *p* < 0.01). On the other hand, the free SO_2_ correlated negatively (−0.608, *p* < 0.05) with yellow and positively (0.782, *p* < 0.01) with red pigment, while the blue proportion of color was not affected, neither by free nor by total SO_2_. It seems that the blue proportion negatively (−0.501, *p* < 0.05) correlates with altitude. On the other hand, a positive correlation was observed between hue (0.853, *p* < 0.01), yellow (0.864, *p* < 0.01), and red (−0.765, *p* < 0.01) proportions of color and alcohol level. An explanation could be that at higher ethanol levels, the solubility of some of the pigments is increased, and the formation of some pigments is made possible.

### 3.3. Variation on Phenolic Content and Antioxidant Capacity

**Figure 2:**
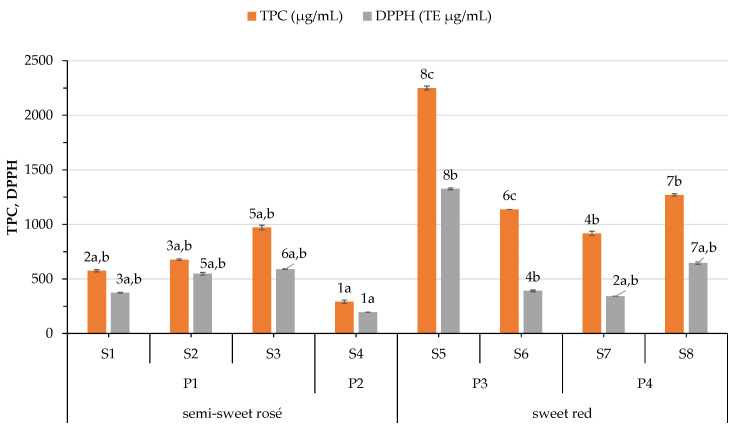
Variation of total phenolic content (TPC) and DPPH antioxidant capacity of wines according to wine type (semisweet rosé and sweet red) and process (P1–P4) applied. Means of samples with any similar superscript number for the same parameter reveal that samples do not differ significantly (*p* < 0.05) by Duncan’s multiple range test. Means of samples with any similar superscript letter for the same parameter reveal that processes do not differ significantly (*p* < 0.05) by Duncan’s multiple range test.

In the present study, the amount of TPC and DPPH antioxidant capacity ranged from 291.63 to 2250.00 μg GAE/mL and 192.23 to 1326.14 μg TE/mL, respectively, revealing differences among the samples (Figure 2). In general, sweet red wines have a higher phenolic content and antioxidant activity than semisweet rosé wines. Paixão et al. [45] observed that red wines have higher TPC and antioxidant levels than white and rosé wines, but observed no difference among the red wines. In general, significant differences observed among the wines in the present study may be attributed not only to the differences in the varietal composition of the wines, but also to the different drying and winemaking processes applied.

Lisov et al. [46] observed that the content of single phenolic compounds (e.g., gallic acid, syringic acid, *p*-coumaric acid, catechin, epicatechin, quercetin) and TPC of wines from fully ripe grapes is higher than from véraison. They noted that single phenolics decreased in wines from overripe grapes while the amount of TPC measured spectroscopically did not. In addition to the winemaking technique, they noted that differences in TPC can be due to inoculation by yeasts, as well as spontaneous fermentation. On the other hand, Costello et al. [47] reported that the phenolic content depends on enzymatic reactions or the metabolic activities of the yeast strain involved.

Indeed, statistical analysis revealed significant differences among the four processes. The drying and winemaking procedure used to produce S3 is characterized by a longer exposure to the sun and a longer spontaneous fermentation compared to that employed in samples S1 and S2. Regarding the sweet red wines, samples S5 and S6 obtained with process P3 exhibited higher values than those (S7 and S8) produced with P4. During P4, must is exposed to longer fermentation time (spontaneous fermentation) and part of it to higher temperatures during its concentration, both affecting the final amount of phenolics.

DPPH antioxidant capacity of wines is closely related to their phenolic content determined by the Folin–Ciocalteu method, as revealed by the very high correlation coefficient (0.919 at *p* < 0.01). Similar conclusions were drawn from Paixão, Perestrelo, Marques, and Câmara [45]. TPC was negatively (−0.603, *p* < 0.05) correlated with free SO_2_ content of wines. In their study, Garaguso and Nardini [48] noted no difference in the polyphenols content, phenolics profile, and antioxidant activity among the wines produced with or without SO_2_.

TPC correlates positively with the intensity and percentage of blue pigment (0.681 and 0.731, *p* < 0.01, respectively), but negatively (−0.667, *p* < 0.01) with the red pigment. On the other hand, DPPH correlated only with blue pigment (0.687, *p* < 0.01). This fact could be linked to the amount of phenolic compounds responsible for the respective color hue in the wines.

### 3.4. Level of Elemental Concentrations in Wines

**Figure 3:**
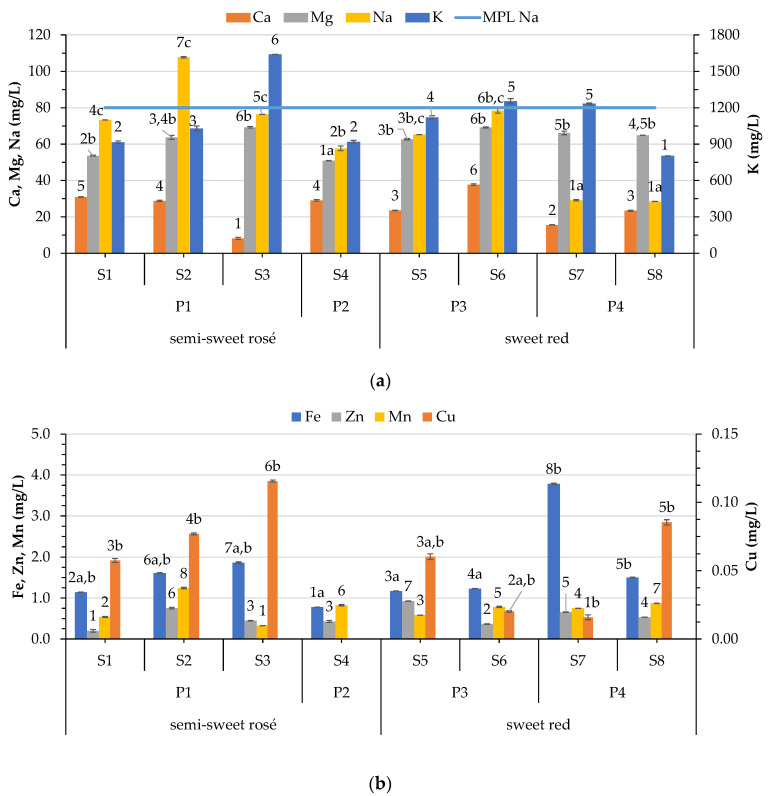
Variation in the amount of macro- and microelements of wines according to the type of wine (semisweet rosé and sweet red) and process (P1–P4): (**a**) macroelement concentration in wine; (**b**) microelement concentration. Values are means of three replicates. Means of samples with any similar superscript number for the same parameter reveal that samples do not differ significantly (*p* < 0.05) by Duncan’s multiple range test. Means of samples with any similar superscript letter for the same parameter reveal that processes do not differ significantly (*p* < 0.05) by Duncan’s multiple range test.

Chemical elements such as Na, Mg, Ca, K, P, S, Cu, Co, I, Zn, and Fe are natural constituents of grapes. The final amount of metals in wine is a result of endogenous (grape variety, soil, and climate) and exogenous (cultivation practices, winemaking processes, and pollution) sources [20]. Elemental analysis in wine with the ICP–OES technique has been used extensively to detect adulteration and determine the authenticity of wines [13,14,15,16,17]. In the present study, the obtained values for the selected metals were compared to the maximum allowable levels at the international level set by OIV standards. The compositions of the wine samples analyzed during this study are presented in Figure 3 and Table 3.

#### 3.4.1. Variation of Macroelement Concentrations in Wines

From the resulting data, K is the main cation of the wines. In this study, the K levels were between 802.27 and 1640.02 mg/L, varying between 916.04 and 1640.02 mg/L in semisweet rosé wines, and 802.27 and 1253.90 mg/L in red wines (Figure 3a). The amount of K in sweet wines from the Canary Islands in Spain (581–1360 mg/L [22]) showed a lower variation than the one reported in this study. Wine samples from other Greek regions presented lower (50.3–123.2 mg/L) [35] or similar (321 ± 2.1–1267 ± 10.0) [14] range levels. In an earlier study, it was reported that potassium values range between 500 and 1300 mg/L [49]. More recent studies report higher variation (152 to 1962 mg/L [50], 97 to 3250 mg/L [51], 271.8 to 1484.0 mg/L [52]). The high variation can be attributed to different factors. Aside from the variety and processing, it can be attributed, to some extent, to the differences among the types of wine (274.10–303.07 mg/L in white wines, 747.22–818.82 mg/L in red wines, and 644.94–748.19 mg/L in rosé wines) [53]. The high levels of K can be increased if there is prolonged contact of the grape skin with the juice or by pressing, as well as by the addition potassium salts during vinification or fining of wines [20]. Nevertheless, there is no significant difference between semisweet rosé and sweet rosé in this study, or among the different winemaking processes adopted. In the study of Đurđić, Pantelić, Trifković, Vukojević, Natić, Tešić, and Mutić [17], it was reported that red wines have higher K values than white. The location also plays a significant role, as revealed by Fabani, Arrúa, Vázquez, Diaz, Baroni, and Wunderlin [50], who reported very high levels of K (1274–1962 mg/L) in one of the locations studied. The amount of K previously found in wine originating from the Aegean islands was 651 to 1093 mg/L [14].

Extreme amounts of potassium, either too low or too high, will lead to a problem in the progression of the fermentation since it slightly impacts the maintenance of cell viability, which links to the arrest of fermentation [54,55]. A high concentration of potassium causes precipitation of free acids (mainly tartaric acid), leading to an increased wine pH. A positive correlation was observed between potassium concentration and pH (0.802, *p* < 0.01) in the present study. Several research studies have found a similar positive correlation [56,57,58]. Kment, Mihaljevič, Ettler, Šebek, Strnad, and Rohlová [58] speculated that this dependence could be related to the ability of bentonites to exchange ions of H+ from wine organic acids.

Together with potassium, calcium is a natural element in must and wine. As in the case of K, Ca is required in the must to ensure normal fermentation. However, the literature reports that elevated levels of Ca can suppress fermentation [59]. Values of Ca in the present study vary from 8.15 to 37.72 mg/L, showing significant differences among the samples (Figure 3a). These values are less than those reported for sweet wines (70–124.3) by Frías, Conde, Rodríguez-Bencomo, García-Montelongo, and Pérez-Trujillo [22]. Nevertheless, no difference was observed in the Ca levels between semisweet rosé and sweet red samples or among the applied processes. The Ca content found is much lower than the levels reported for wines of the Aegean islands (159–539 mg/L) [14]. The literature reports similar or higher values, and a higher range of variation (22.19–65.78 mg/L [53], Ca (32 to 137 mg/L) [51], 29.9–114.8 mg/L [52]). Bora, Călugăr, Bunea, Rozsa, and Bunea [53] reported that rosé (22.19–32.47 mg/L) wines showed the lowest Ca levels followed by red (42.51–47.71 mg/L) and white (60.95 and 65.78 mg/L) wine samples. On the other hand, Đurđić, Pantelić, Trifković, Vukojević, Natić, Tešić, and Mutić [17] reported no difference among the red and white samples in the amount of Ca. Moreover, they reported significant variation among the vineyards and winemaking practices (organic, conventional, and homemade). A negative correlation was observed between Ca and pH (−0.502, *p* < 0.05) and the alcohol strength of wines (−0.585, *p* < 0.05).

Birch, Ciani, and Walker [59] reported that high calcium levels interfere with the uptake of magnesium from yeast cells. In this study, magnesium levels vary from 50.91 to 69.25 mg/L (Figure 3a), being lower than sweet wines from the Canary Islands (64.3–135.4 mg/L) [22]. These values are within the range reported in the literature (42.7–161 mg/L [51], 23.8–160.8 mg/L [52]). Contrarily, higher levels were observed in Romanian wines (90.38–132.21) [53], which noted differences among the types of wines (90.38–114.78 mg/L for white, 100.71–105.07 mg/L for red, and 98.63–132.21 mg/L for rosé wines). Sweet red wines in this study exhibited significantly higher values than semisweet rosé wines. Đurđić, Pantelić, Trifković, Vukojević, Natić, Tešić, and Mutić [17] reported higher Mg values in red than in white wines of Serbia. In the present study, it was observed that vinification significantly affected the final amount of Mg in the studied wines, with P2 leading to the lowest levels compared to the rest of the processes (P1, P3, P4) that showed no difference among them. The literature reports that the applied vinification process (commercial or homemade) significantly affects the levels of Mg [53]. Correlation analysis revealed that Mg significantly correlates with the amount of free SO_2_ (−0.810, *p* < 0.01), alcoholic strength (0.573, *p* < 0.05), as well as color parameters: intensity (0.597, *p* < 0.05), hue (0.712, *p* < 0.01), %yellow (0.639, *p* < 0.01), and %red (−0.833, *p* < 0.01).

The amount of Na significantly differs among the samples, having a mean ranging between 28.43 and 78.32 mg/L in sweet red samples, and 57.60 and 107.73 mg/L in semisweet rosé samples (Figure 3a). Sodium content in wines derives from natural (sea proximity) or artificial sources (e.g., fining, clarifying agents, and different salts used as additives/preservatives) [20]. In particular, the vineyard location next to the sea plays a crucial role [22] in the final amount of Na. One semisweet rosé sample (S2) had a Na level (107.73 mg/L) above the one set by OIV (80 mg/L). This sample, together with sample S1, derives from vineyards of an altitude of 35 m in the direction from north to south, where the saltiness and the sea breeze have a permanent effect on them. Relative high values of samples S1, S2, S3, and S6 can be explained by the lower altitudes of these vineyards and their proximity to the sea.

Greek wines showed a high variation of sodium depending on the vineyard location (2.7–187 mg/L) [14]. Significantly lower Na concentrations for white and red wines from continental Greece (Drama region) [35] were observed, while in wines originating from the Aegean islands, higher levels were noted (55.2 to 187 mg/L) [14]. Díaz et al. [60] reported values in the range of 10–310 mg/L for red wines from Canary Islands, Spain, and attributed the high levels to the influence of the marine spray. This fact is supported by other studies on sweet wines from different regions of the Canary Islands and diverse years that also report high values of sodium (164.4 ± 29.3 and 79 ± 34 [22], and 50–209 mg/L) [23]. In another study, the corresponding values were 75.4 ± 16.5 mg/L in the wines of the region Biscoitos of the Azores, Portugal [61]. In an early study, a mean value of 88 mg/L was reported for sodium in Californian wines [49]. Portuguese wines, red and white, also showed relatively high values (4.53–92.2 mg/L [52]). The effects of sea spray are more pronounced on the islands or the coasts than on the continent. The amount of Na in wines from wine regions from other countries is much lower (Serbia 3.07–37.05 mg/L [17], Poland 5.33 µg/L to 3.823 mg/L [51], Czechia 2–62 mg/L) [58].

Na levels in the present study were higher in semisweet rosé wines than in sweet red wines. In their study, Đurđić, Pantelić, Trifković, Vukojević, Natić, Tešić, and Mutić [17] reported that red wines were lower in Na than white wines. It seems that the applied vinification process affects the amount of Na in the final product. It is obvious that wines obtained by P4 have the lowest level of Na, followed by P2, which does not differ from wines produced by P3. Finally, the wine samples produced with P1 show the highest values. In this process, fermentation was stopped artificially by decreasing the temperature, whereas in the other three processes this occurred spontaneously. However, this difference can be attributed to the low altitude in the samples processed with P1. The amount of Na correlates negatively with the hue (−0.658, *p* < 0.01) and yellow proportion (−0.687, *p* < 0.01) of color.

#### 3.4.2. Variation of Microelement Concentrations in Wines

The variation in the amount of microelements Fe, Cu, Zn, and Mn among the wine samples are reported in Figure 3b. The amount of Fe, Cu, Zn, and Mn among the samples vary from 0.78 to 3.78 mg/L, from <LOQ to 0.12, from 0.2 to 0.93 mg/L, and from 0.33 to 1.24 mg/L, respectively. These values are similar to Fe values (0.8–3.6 mg/L), less than Cu (0.09–0.62) and Zn (0.16–1.52) values, and slightly higher than Mn (0.52–0.96) values reported for sweet wines [22]. All of the wines studied had Fe, Cu, and Zn contents below 20 mg/L, 1 mg/L, and 5 mg/L, respectively, as considered by the OIV maximum permissible limits. Moreover, these levels are far lower than the concentrations that create cloudiness or iron and copper cases [20]. Although the concentration of microelements in wine is low, they have a catalytic role in yeast fermentation. Mn, together with Fe and Cu, affect wine oxidation and, in addition, they favor chemical processes that form acetaldehydes [62].

Compared to Greek samples from the Aegean Islands, the values reported here have much lower Fe, Cu, and Zn contents, and are of similar or lower Mn values [14]. Differences were also observed in the literature data reporting microelements in international wines. Fe and Cu levels vary among the countries: Portugal (0.04–0.38 and 0.001–0.043 mg/L, respectively) [52], Poland (0.1558–2.775 and 0.0002–0.796 mg/L) [51], Serbia (0.01–6.01 mg/L and 0.024–1.684 mg/L, respectively) [17], Romania (0.66–1.79 and 0.14–0.55 mg/L mg/L, respectively) [53].

Variations in our results can be attributed to differences introduced by factors such as type of wine, location, and variety [17,53]. The wines do not differ (in Fe levels) on the base of their type (semisweet rosé, sweet red), but differ in the process applied; P2 and P3 do not differ between them, and with P1, but differ with P4. Bora, Călugăr, Bunea, Rozsa, and Bunea [53] reported that Fe concentrations differ based on location, but were similar among the same wine types and culture systems.

The Mn and Zn values present in this study are higher than Portuguese wines reported by Rocha, Pinto, Almeida, and Fernandes [52] (0.02–0.40 mg/L and 0.012–0.0723 mg/L, respectively), but lower than Polish, Serbian, and Romanian wines examined by Płotka-Wasylka, Frankowski, Simeonov, Polkowska, and Namieśnik [51] (0.329–9.219 mg/L and 0.075–2.339 mg/L, respectively), Đurđić, Pantelić, Trifković, Vukojević, Natić, Tešić, and Mutić [17] (0.294–3.310 mg/L and 0.215–2.585 mg/L, respectively), and Bora, Călugăr, Bunea, Rozsa, and Bunea [53] (0.38–0.96 mg/L and 1.830 ±3.289 mg/L, respectively).

Although high Zn levels in wine are attributed to different sources (soil, fungicides/insecticides, irrigation, equipment) [20], Bora, Călugăr, Bunea, Rozsa, and Bunea [53] noted that the blame is on the excessive use of fungicides and insecticides for values near the limit set by OIV. It seems that neither the wine type nor the process affect the Mn and Zn levels in the studied wines. Similarly, Bora, Călugăr, Bunea, Rozsa, and Bunea [53] reported that the mean Zn concentrations were comparable between wine types and culture systems. The literature reports that the final levels of Cu and Zn can be significantly decreased depending on bentonite fining [63].

No differences were observed between sweet red and semisweet rosé wines for the microelements in the present study. In their study, Đurđić, Pantelić, Trifković, Vukojević, Natić, Tešić, and Mutić [17] reported higher levels of Fe and Mn in red than in white wines, while the opposite was observed for Cu [17,53] and Zn [17]. Although in the literature it was reported that Fe and Cu have a great complexing capacity towards natural polyphenols [64], no correlation was observed. Yet, a significant correlation at (*p* < 0.01) was observed between Zn and TPC (r = 0.628) and DPPH (r = 0.706).

The literature reports that Fe, Cu, and Mg have complexing abilities with anthocyanins and tannins, resulting in wine color alteration [65]. Moreover, Fe and Cu were reported to increase the browning rates of phenolics (i.e., quercetin and rutin) [66]. In the present study, besides correlations of metals with color parameters of wines mentioned previously, there is observed a significant correlation of Fe with hue (0.733 *p* < 0.01) and yellow proportion of color (0.758, *p* < 0.01) and red (−0.580, *p* < 0.05), though no other microelement showed significant correlations.

#### 3.4.3. Variation of Trace Element Concentrations in Wines

As previously reported, factors affecting the concentrations of toxic metals in wine are chemicals used in viticulture, oenological products, equipment, environmental contamination, and poor cellar practice. Among the trace elements, arsenic was not detected in our samples. High levels of Pb and Cd are mainly associated with environmental pollution (proximity of vineyard to road traffic or industrial areas) [20,67]. In addition, long contact of grape/wine with various pieces of equipment containing alloy metals is considered a contamination source [20]. The results showed that Cd and Pb levels in samples S3 and S6 were lower than the limit (0.01 mg/L and 0.015 mg/L, respectively). On the other hand, in the rest of the samples, Cd and Pb could not be detected (below LOD). The maximum values in the work of Skendi, Papageorgiou, and Stefanou [35] showed slightly higher Cd values. Cd and Pb values vary largely in the literature (0.023–2.54 µg/L) [51], (3.41–65.4 µg/L) [52], (0.07–11.1 µg/L) [17] and 0.14−1.16 µg/L [52], 9.3–188.2 µg/L [17], respectively. In another study, authors reported mean values of 0.39 µg/L for Cd and 32.35 µg/L for Pb [53].

The amount of Cr and Ni varies among the samples (Table 3). Some samples showed Cr values lower than the limit of detection, while three of them (S2, S3, and S5) have less than 1.95 μg/L. The Ni values vary from 79.90 to 120.4 μg/L. The Cr amount is much lower than that reported in the literature [17,51,52,53,68]. On the other hand, Ni levels were higher than those reported by Rocha, Pinto, Almeida, and Fernandes [52] (0.63–3.76 μg/L) and Bora, Călugăr, Bunea, Rozsa, and Bunea [53] (31.86–72.65 μg/L), but lower than the results of Płotka-Wasylka, Frankowski, Simeonov, Polkowska, and Namieśnik [51] (0.02–245 μg/L) and Alkış, Öz, Atakol, Yılmaz, Anlı, and Atakol [68] (45.29–3605.65 μg/L). Generally, Ni and Cr can migrate from the metal equipment used in winemaking to the final wine. The acidity of wine can favor this migration. In addition to the low levels of pollution, our results reveal that good winemaking practices were followed by the producers, preventing this migration.

The literature reports that in polluted areas, the concentrations of heavy metals (Cr, Mn, Ni, Cd, Pb, and As) were found in higher levels in grape skins than in the pulps [69], and thus higher in red wines that remain in high contact with the skin. Nevertheless, in the present study, there is no significant difference in the levels of Cd, Pb, Ni, and Cr between sweet red and semisweet rosé wines. Controversy exists among researchers in the literature regarding the levels of trace metals in different types of wines. Bora, Călugăr, Bunea, Rozsa, and Bunea [53] compared white, red, and rosé samples for their Cr and Ni concentrations, and reported that these values tended to be higher in white wine samples, followed by red and rosé samples.

Alkış, Öz, Atakol, Yılmaz, Anlı, and Atakol [68] agree regarding Ni concentration in wines, but do not observe differences in the Cr amount among types of wines. On the contrary, Rocha, Pinto, Almeida, and Fernandes [52] reported that concentrations of Cr and Ni were higher in red wines, while Pb and Cd were higher in white wines. Đurđić, Pantelić, Trifković, Vukojević, Natić, Tešić, and Mutić [17] reported that white wines have higher Pb and Ni levels than red ones, while the opposite was observed for Cr. Moreover, they did not observe differences in Cd levels between the red and white wines. Rodrigues, Otero, Alves, Coimbra, Coimbra, Pereira, and Duarte [15] reported higher levels of Ni in red wine samples when compared with white wine.

When wines were considered by the winemaking process, Cd, Pb, and Cr were present at similar concentrations among the four processes applied, except for Ni. The amount of Ni increases in the following order P2 < P1 = P4 < P3.

### 3.5. Chemometric Assessment of Wines

**Figure 4:**
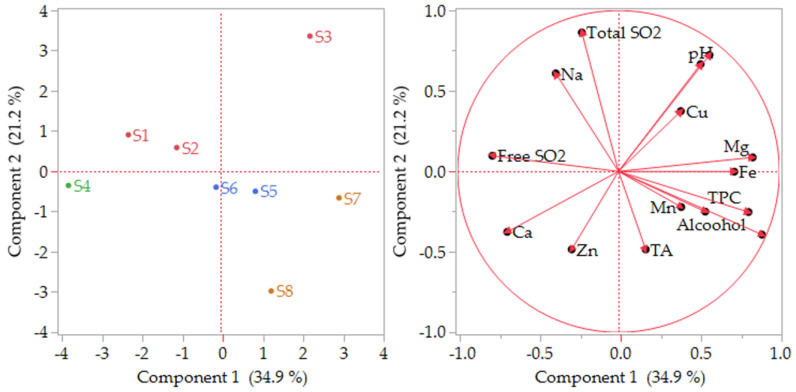
Principal component analysis (PCA) showing sample scores obtained analyzing the wine samples from Kos Island (**left**) and variables (**right**) on the first two principal components.

The chemometric method employed to analyze the data was principal components analysis (PCA). All variables were included in the analysis, except those highly correlated (i.e., wine intensity, DPPH, %yellow, %red, %blue). PCA was performed to reduce the number of variables and detect relationships among them. Five principal components were considered significant (eigenvalue higher than 1), accounting for 91.4% of the total variance. The first two factors (PC1 and PC2) represent 56.2% of the initial data variability, and 71.7% with the third factor (PC3). Figure 4 (left) shows sample scores arranged based on the two factors PC1 and PC2. Sweet red samples were grouped at the negative values of PC2. Samples S5 and S6 are close to each other, suggesting similarities confirmed by the same process applied to these wines. To the contrary, samples S6 and S7, although belonging to the same processing type, are distant from each other. This difference can be explained by the fact that these wines are produced by a spontaneous must fermentation. Granchi et al. [70] reported that biodiversity in spontaneous fermentation results in different fermentations in the same winery, resulting in differing wines.

The semisweet rosé samples are spread in three quadrants. Samples S1, S2, and S3 (belonging to the vinification process P1) were grouped together and are located at the positive values of PC2. At the negative values of PC1 are located all the sun-dried samples S1 and S2 from P1 process, together with S4 from P2. At the positive values of PC2, there are located samples from the P1 process (S1, S2, and S3), where fermentation was stopped by cooling. In conclusion, the scatter plot of PCA scores shows that wine samples were clearly separated according to the processing procedure (P1, P2, P3, P4) applied. The variables describing this grouping of sweet wines are TA, alcohol, TPC, hue, Mn, and Fe, as noted in Figure 4 (right), suggesting that these parameters are the main ones responsible for this grouping.

## 4. Conclusions

Four sweet red and four semisweet rosé wines from Kos Island were characterized, and their composition was discussed based on the winemaking process followed in their production. The studied wine samples, especially the red ones, are distinguished by higher intensity, hue, and higher yellow and red percentages than the rosé ones. Sweet red wine samples had higher alcohol content, total phenolic content, antioxidant activity, and color intensity as compared to semisweet rosé counterparts. The process applied was found to significantly affect the color parameters. In general, a shift towards yellow-brown color in the case of red sweet wines, especially of those produced by increasing sugar level by concentration, was observed. When wines were considered by wine type, most chemical elements (macro-, micro-, and trace elements) were present at similar concentrations in sweet red and semisweet rosé. Only Na levels differed between the two groups, with semisweet rosé wines having higher levels than the sweet red wines; in the case of Mg, the opposite was observed. Altitude is a factor that is negatively correlated with Na content in the studied samples. The wines of the present study appeared to be safe regarding the risk associated with potentially toxic metal intake and the content of SO_2_. The chemometric analysis clearly separated wine samples according to the processing procedure applied. The present is a preliminary study that contributes to the understanding of the quality parameters of sweet wines from Kos Island in Greece. Nevertheless, further studies are needed to evaluate the reported winemaking procedures in order to maintain a high quality in sweet wine production, especially in the context of climate change.

## Figures and Tables

**Table 1:** Description of the wine samples tested in relation to the variety, location, treatment, and production process.

Type of Wine	Sample Code	Varietal Composition	Altitude	Treatment after Grape Harvest	Addition of SO_2_	Production Process
Semisweet rosé	S1	Muscat–Moschofilero	35	Grapes sun-dried for 1 week until ~18–19° Baume	Yes	Inoculation of must in tank. Fermentation is stopped by cooling to 9 °C for a few days.
	S2	Muscat–Merlot	35		Yes	
	S3	Tempranillo	25	Harvesting when grapes reach ~17° Baume (vine-dried grape)	Yes	Inoculation of must with yeasts in tank. Fermentation is stopped by cooling to 0 °C.
	S4	Assyrtiko–Syrah	110	Grapes sun-dried for 10 days until ~20–22° Baume	Yes	Spontaneous fermentation of the must. Fermentation stops due to the effect of osmotic pressure.
Sweet red	S5	Syrah	45	Harvesting when grapes reach ~14–14.5° Baume	Yes	Inoculation of pressed grapes. Added thyme honey (1 kg honey/10 L of wine).
	S6	Merlot–Syrah	45		Yes	
	S7	Sultana–Syrah (40–60%)	100	Sultana traditional way without prior sampling The rest of grapes are harvested at 15.6–16.7° Baume	No	Part of Sultana must (S6)/Merlot must (S7) is boiled until 50% of the initial volume (“Κρύο -ψήμα” cold cooked must, CCM). Addition of CCM in the Syrah must until 18–18.5° Baume (S6)/the rest of grapes up to 18–22° Baume (S7). Spontaneous fermentation of the must. Fermentation stops due to the effect of osmotic pressure.
	S8	Moschofilero (50%) Merlot (40%) Syrah (5%) Cabernet franc (5%)	80		No	

**Table 2:** Concentration (mean ± standard deviation) of enological parameters analyzed in rosé and red wines ^1^.

			pH	Total Acidity (Tartaric Acid g/L)	Free SO_2_ (mg/L)	Total SO_2_ (mg/L)	Alcohol Strength (vol. %)
Type of Wine	Process	Sample	Mean ± SD	Mean ± SD	Mean ± SD	Mean ± SD	Mean ± SD
Semi-sweet rosé	P1	S1	3.56 ± 0.05 ^3a^	5.55 ± 0.01 ^3a^	25.60 ± 0.42 ^6b^	130.45 ± 0.78 ^7c^	12.70 ± 0.14 ^2a^
	P1	S2	3.70 ± 0.00 ^5a^	4.82 ± 0.05 ^1a^	10.25 ± 0.07 ^4b^	73.75 ± 0.92 ^4c^	12.00 ± 0.14 ^1a^
	P1	S3	3.91 ± 0.01 ^6a^	6.25 ± 0.08 ^5a^	16.10 ± 0.00 ^5b^	192.30 ± 0.71 ^8c^	14.00 ± 0.16 ^4a^
	P2	S4	3.35 ± 0.01 ^2a^	6.02 ± 0.05 ^4a^	64.60 ± 0.14 ^7c^	97.95 ± 0.78 ^6b,c^	12.00 ± 0.07 ^1a^
Sweet red	P3	S5	3.57 ± 0.01 ^3a^	5.50 ± 0.04 ^3a^	6.80 ± 0.00 ^2a^	42.55 ± 0.35 ^3a,b^	13.00 ± 0.07 ^3a^
	P3	S6	3.62 ± 0.01 ^4a^	7.49 ± 0.04 ^6a^	7.30 ± 0.42 ^2a^	76.20 ± 0.85 ^5a,b^	13.00 ± 0.06 ^3a^
	P4	S7	3.96 ± 0.01 ^6a^	5.33 ± 0.01 ^2a^	2.95 ± 0.21 ^1a^	15.60 ± 0.28 ^1a^	14.00 ± 0.06 ^4b^
	P4	S8	3.19 ± 0.02 ^1a^	8.38 ± 0.06 ^7a^	9.55 ± 0.21 ^3a^	29.55 ± 0.64 ^2a^	14.80 ± 0.03 ^5b^

^1^ Values are means of three replicates. Means of samples with any similar superscripts number in the same column reveal that samples do not differ significantly (*p* < 0.05) by Duncan’s multiple range test. Means of samples with any similar superscript letter in the same column reveal that processes do not differ significantly (*p* < 0.05) by Duncan’s multiple range test.

**Table 3:** Concentration (mean ± standard deviation) of elements in analyzed sweet, red, and semisweet rosé wines ^1^.

			Cd (μg/L)	Pb (μg/L)	Ni (μg/L)	Cr (μg/L)
Type of Wine	Process	Sample	Mean ± SD	Mean ± SD	Mean ± SD	Mean ± SD
OIV Maximum Permissible Limit (μg/L)			10	150	-	-
Semisweet rosé	P1	S1	<LOD	<LOD	102.92 ± 2.13 ^4b^	<LOD
	P1	S2	<LOD	<LOD	88.59 ± 1.59 ^2b^	1.95 ± 0.17 ^2^
	P1	S3	7.76 ± 0.01 ^2^	53.22 ± 2.09 ^2^	103.34 ± 2.88 ^4b^	0.78 ± 0.18 ^1^
	P2	S4	<LOD	<LOD	79.90 ± 1.05 ^1a^	<LOD
Sweet red	P3	S5	<LOD	<LOD	118.81 ± 1.54 ^5c^	1.83 ± 0.19 ^2^
	P3	S6	1.08 ± 0.29 ^1^	32.02 ± 0.79 ^1^	120.40 ± 3.02 ^5c^	<LOD
	P4	S7	<LOD	<LOD	97.81 ± 2.40 ^3b^	<LOD
	P4	S8	<LOD	<LOD	87.75 ± 2.02 ^2b^	<LOD

^1^ The results are means of three replicates ± standard deviation. <LOD (limit of detection): values lower than the limit of detection. For each element, different numbers as superscripts indicate significant differences between samples (*p* ≤ 0.05) as determined by Duncan’s multiple range test. Means of samples with any similar superscript letter in the same column reveal that processes do not differ significantly (*p* < 0.05) by Duncan’s multiple range test.

## Data Availability

Data are contained within the article.
